# Phosphodiesterase 9A in Brain Regulates cGMP Signaling Independent of Nitric-Oxide

**DOI:** 10.3389/fnins.2019.00837

**Published:** 2019-08-23

**Authors:** John F. Harms, Frank S. Menniti, Christopher J. Schmidt

**Affiliations:** ^1^Internal Medicine Research Unit, Pfizer Global Research and Development, Cambridge, MA, United States; ^2^George & Anne Ryan Institute for Neuroscience, The University of Rhode Island, Kingston, RI, United States; ^3^Pfizer Innovation and Research Lab Unit, Pfizer Global Research and Development, Cambridge, MA, United States

**Keywords:** cGMP, PDE9A, phosphodiesterase inhibitor, nitric oxide, nitric oxide synthase, brain, cognitive disorders

## Abstract

PDE9A is a cGMP-specific phosphodiesterase expressed in neurons throughout the brain that has attracted attention as a therapeutic target to treat cognitive disorders. Indeed, PDE9A inhibitors are under evaluation in clinical trials as a treatment for Alzheimer’s disease and schizophrenia. However, little is known about the cGMP signaling cascades regulated by PDE9A. Canonical cGMP signaling in brain follows the activation of neuronal nitric oxide synthase (nNOS) and the generation of nitric oxide, which activates soluble guanylyl cyclase and cGMP synthesis. However, we show that in mice, PDE9A regulates a pool of cGMP that is independent of nNOS, specifically, and nitric oxide signaling in general. This PDE9A-regulated cGMP pool appears to be highly compartmentalized and independent of cGMP pools regulated by several PDEs. These findings provide a new foundation for study of the upstream and downstream signaling elements regulated by PDE9A and its potential as a therapeutic target for brain disease.

## Introduction

PDE9A is a phosphodiesterase specific and with high affinity for cGMP. This enzyme is coded by a single gene but is expressed as more than 20 isoforms arising from mRNA alternative splicing (Fisher et al., 1998; Rentero et al., 2003; Kotera and Omori, 2006). PDE9A mRNA is detected throughout the body and notably in brain (Lakics et al., 2010; Kelly, 2012). In brain, *in situ* hybridization studies indicate PDE9A mRNA is primarily neuronal with highest levels in Purkinje neurons in cerebellum, and significant levels in cortex, the pyramidal cell layer of the hippocampus, and throughout the striatum (Andreeva et al., 2001; van Staveren and Markerink-van Ittersum, 2005). Several classes of PDE9A inhibitors have been developed (Wunder et al., 2005; Verhoest et al., 2012; Su et al., 2016; Boland et al., 2017) and used to investigate PDE9A physiology in brain. In primary cultures of rat hippocampal neurons, PDE9A inhibition enhanced neurite outgrowth and the number of synapses per neurons (Hutson et al., 2011). Two groups have reported that PDE9A inhibition facilitates induction of long-term potentiation at CA3/CA1 synapses in acute hippocampal slices (Hutson et al., 2011; Kroker et al., 2012, 2014). Systemic administration of PDE9 inhibitors to rodents has been shown to increases regional concentrations of cGMP in brain and CSF, and the elevation of cGMP in rodent CSF has now been translated to healthy humans by two groups (Schmidt et al., 2009; Boland et al., 2017). Notably, PDE9A inhibitors improve performance in several animal models of cognitive function (van der Staay et al., 2008; Hutson et al., 2011; Vardigan et al., 2011; Kleiman et al., 2012; Alexander et al., 2016). Based on these finding, PDE9A inhibitors have been advanced into initial clinical studies to assess the potential to improve cognitive function in patients with Alzheimer’s disease and schizophrenia (Schwam et al., 2014; Wunderlich et al., 2016; Boland et al., 2017). More recent studies have demonstrated that PDE9A inhibition also ameliorates auditory gating deficits in the BACHD transgenic rat model of Huntington’s disease (Nagy et al., 2015), and reduces a social withdrawal deficit in dystrophin-deficient mice, a model of an autism-like behavioral deficit associated with Duchenne muscular dystrophy (Alexander et al., 2016). Thus, it appears that PDE9A-regulated cGMP signaling has important neuronal function(s) and PDE9A inhibitors may have therapeutic utility in treating a range of neuropsychiatric disorders.

There is as of yet little information on the molecular pathways that mediate the reported effects of PDE9 inhibitors on synaptic plasticity and behavior. In immunohistochemical analyses, PDE9-like immunoreactivity is detected in cell bodies and proximal dendrites of Purkinje neurons, cortical pyramidal neurons, and neurons in subiculum (Kleiman et al., 2012; Patel et al., 2018) but not in more distal neuronal processes. Recently, it was reported that PDE9A splice variants are localized to membrane and nuclear fractions upon subcellular fractionation of mouse cerebellum and hippocampus (Patel et al., 2018). It is not known whether these findings indeed reflect a restricted protein distribution or failure to detect the full scope of the distribution due to the lack of specific high affinity antibodies and/or very low levels of protein expression. Nonetheless, at this point PDE9A has not been placed at synapses. There is also little information on the signaling mechanisms that drive PDE9A-regulated cGMP signaling. cGMP is produced by either particulate guanylyl cyclase (pGC) or soluble guanylyl cyclase (sGC) (Castro et al., 2006). The pGC is present at the cell membrane and is activated by natriuretic peptides, whereas sGC is present in the cytosol and is activated by nitric oxide (NO). NO has an established role in neuronal signaling that continues to be very actively studied (Garthwaite, 2008). Natriuretic peptides and their receptors are also widely expressed in the brain (Cao and Yang, 2008), but these signaling cascades are less well characterized. Recently, it was reported that PDE9A exclusively regulates pGC cGMP signaling in cardiac tissue (Lee et al., 2015; Kokkonen and Kass, 2017). However, there are no published data to indicate whether pGC or sGC is driving the PDE9A-regulated cGMP cascade in brain.

Identifying the cGMP signaling pathway regulated by PDE9A in brain is critical to understanding the neurophysiological functions of this phosphodiesterase and its potential as a therapeutic target. In the studies presented here, we begin to investigate this question by determining the effects pharmacological agents and phosphodiesterase gene knock outs on PDE9A inhibitor-induced elevations of cGMP in different brain regions in mouse. Our results indicate that PDE9A regulates discreet cGMP signaling cascades that, as reported in cardiac tissue, are independent of NO signaling.

## Materials and Methods

### PDE9A Inhibitors

Two structurally distinct inhibitors from Pfizer’s PDE9A medicinal chemistry program were used in these studies: PF-509783 (compound 2) and PF-4181366 (compound 19) from Verhoest et al. (2009). The selectivity of these two compounds for PDE9A inhibition over inhibition of other phosphodiesterases was determined as previously described (Schmidt et al., 2008; Verhoest et al., 2009). PF-4181366 is highly potent and selective for PDE9A (PDE9A IC_50_ = 2 nM; PDE1C IC_50_ = 73 nM; PDE9A selectivity vs. PDE1C and all other PDEs > 36-fold). An early compound, PF-509783 is less potent and selective (PDE9A IC_50_ = 26 nM; PDE1A IC_50_ = 8 nM; PDE1C IC_50_ = 8 nM; PDE5A IC_50_ = 42 nM; PDE6A IC_50_ = 34 nM). However, the effect of PF-509783 on brain cGMP levels was completely occluded in *Pde9a* knock out mice (Table 1, italicized), demonstrating the functional selectivity of PF-509783 for PDE9A inhibition with regard to brain cGMP responses. PDE9A inhibitors were administered in a vehicle of 5% DMSO, 5% cremophor, 90% saline.

**TABLE 1:** PDE9A inhibitor-induced increases in cGMP in wildtype vs. knockout mice or in the absence vs. presence of glutamate or dopamine modulators.

	**Striatum**				**Hippocampus**			
**PF-509783**	**Ave.**	**SEM**	**Ave.**	**SEM**	**Ave.**	**SEM**	**Ave.**	**SEM**
				
	**Wild type**		**Knockout**		**Wild type**		**Knockout**	
*PDE9A*	*0.061*	*0.03*	− 0 < /.007	*0.01*	*0.047*	*0.02*	− 0. 019	*0.02*
PDE1b	0.095	0.018	0.098	0.025	0.090	0.019	0.066	0.021
PDE10A	0.089	0.009	0.084	0.011	0.065	0.014	0.051	0.013
**PF-4181366**								
n-nos	0.102	0.003	0.096	0.003	0.063	0.009	0.048	0.004
e-nos	0.098	0.014	0.095	0.010	0.055	0.016	0.059	0.017
i-nos	0.113	0.014	0.064	0.018	0.066	0.016	0.041	0.008

**PF-509783**	**Vehicle**		**Drug**		**Vehicle**		**Drug**	

MK-801	0.056	0.008	0.059	0.014	0.061	0.014	0.052	0.014
CP-465022	0.078	0.012	0.049	0.008	0.042	0.010	0.030	0.010
SCH-23390	0.089	0.008	0.064	0.007	0.042	0.008	0.035^∗^	
Haloperidol	0.076	0.016	0.053	0.019	0.083	0.036	0.063	0.014
**PF-4181366**								
WIN 55212^∗∗^	0.100	0.006	0.084	0.008	0.060	0.009	0.036	0.011

*First, for each condition the mean cGMP level in the absence of PDE9A inhibitor was calculated and defined as basal level (*n* = 5). Then, the condition-specific basal value was subtracted from each condition-specific sample from PDE9A inhibitor treated subjects to determine an increase in cGMP over “basal” level caused by PDE9A inhibition. These calculated values were averaged and expressed in the table as mean ± standard error of the mean (column headings Ave. and SEM). *Italics*: The effect of PF-509783 to increase cGMP levels in wild type mice was completely abrogated in *Pde9a* knock out mice. Each value represents data from *n* = 5 animals unless noted by ^∗^, in which case *n* = 4, or noted by ^∗∗^, which case *n* = 6.*

### Other Drugs

MK-801 and SCH-23390 were administered in saline, and haloperidol in aqueous 0.3% tartaric acid (Sigma-Aldrich, St. Louis, MO, United States). WIN 55212 was dissolved in the same vehicle used for the PDE9A inhibitors. CP-465,022 (Menniti et al., 2003) was administered in 40% (2-Hydroxypropyl)-β-cyclodextrin (synthesized by Pfizer, Inc.).

PDE9A inhibitors were administered 30 min prior to sacrifice in all studies. For pharmacological interaction studies, SCH-23390, MK-801, CP-465022 or WIN 55212 were administered 20 min prior to PF-509783 or PF-4181366. Haloperidol was administered 5 min after PF-509783 and the animals were sacrificed after a further 25 min. All drugs were administered subcutaneously (s.c.) in a volume of 10 ml/kg.

### Animals

Male CD-1 mice 6–8 weeks of age were obtained from Charles River Breeding Laboratories. *Pde9a* global knockout mice were developed by Pfizer Inc. and described in detail by Lee et al. (2015). *Pde10a* global knockout mice were also developed by Pfizer Inc. and described in detail by Siuciak et al. (2006b, 2008). *Pde1b* global knockout mice were developed by Reed et al. (2002). The mice used in these experiments were from a colony established and maintained at Charles River Laboratories (CRL, Wilmington, MA, United States) and described in detail by Siuciak et al. (2007). Neuronal nitric oxide synthase (*nNOS*) (Siuciak et al., 2006a), endothelial nitric oxide synthase (*eNOS*) (Shesely et al., 1996), and inducible nitric oxide synthase (*iNOS*) (Laubach et al., 1995) knockout mice were from Jackson labs (JAX stock # 002986, 002684, and 002609, respectively). Wild-type C57BL/6J mice were used as controls as suggested by the vendor. All animals were housed under standard laboratory conditions on a 12-h light/dark cycle. Food and water were provided *ad libitum* and animals were acclimated to the vivarium for at least 5–7 days before experimentation. Animals were handled and cared for according to the Guide for the Care and Use of Laboratory Animals (Institute of Laboratory Animal Resources, 1996) and the Pfizer Institutional Animal Care and Use Committee approved all procedures.

Measurements of cGMP accumulation in brain tissues were performed essentially as described previously (Schmidt et al., 2008). Mice were euthanized by focused microwave irradiation of the forebrain after administration of vehicle or pharmacological agents (*n* = 5 animals per treatment group unless otherwise noted). Animals were housed 4–5 per cage and each treatment group was represented at least once per cage. Regions of interest were isolated, homogenized in a lysis buffer containing: 100 mM NaCl, 10 mM Tris, pH 7.4, 1 mM EDTA and 0.3% NP-40. Aliquots were acidified with 1 part 6N HCl to 10 parts homogenate, and centrifuged. Supernatant concentrations of cGMP were measured using enzyme immunoassay kits (Cayman Chemical, Ann Arbor, MI, United States). Experimental groups were distributed across the assay plate to avoid edge or other systematic effects.

### Study Design and Analysis

The primary measure for all studies reported here was the concentration of cGMP in individual brain regions. We interrogated how these levels changed in response to PDE9A inhibition alone or in the context of a second manipulation that would also affect cGMP turnover using a Latin-square design. The sample sizes of *N* = 5 were selected based on prior experience with the determination of cGMP concentrations in rodent brain using either genetic or pharmacological manipulations to increase or decrease tissue cGMP. Reported results are from a single experiment unless noted otherwise. Statistical analyses of individual experiments entailed a 2-way ANOVA using GraphPad Prism (version 6.03), with *post hoc* tests including a Bonferroni correction factor for multiple comparisons. Based on the robust nature of the *F* statistic for identical group sizes with equivalent variance we did not test for normality. In some experiments, the lack of a significant interaction term in the 2-way ANOVA was interpreted to indicate that the effect of PDE9A inhibition was independent of the effect of the second manipulation. Data are also presented as the absolute PDE9A inhibitor-induced increase in cGMP over a “basal” level. This was calculated as the pMol increase of cGMP/mg tissue produced by PDE9A inhibition in vehicle-treated versus drug-treated or wild type versus transgenic mice (see Table 1). Data are available upon request.

## Results

### Effects of PF-509783, PF-4181366, and PDE9 Knock Out on Brain cGMP Levels

**FIGURE 1:**
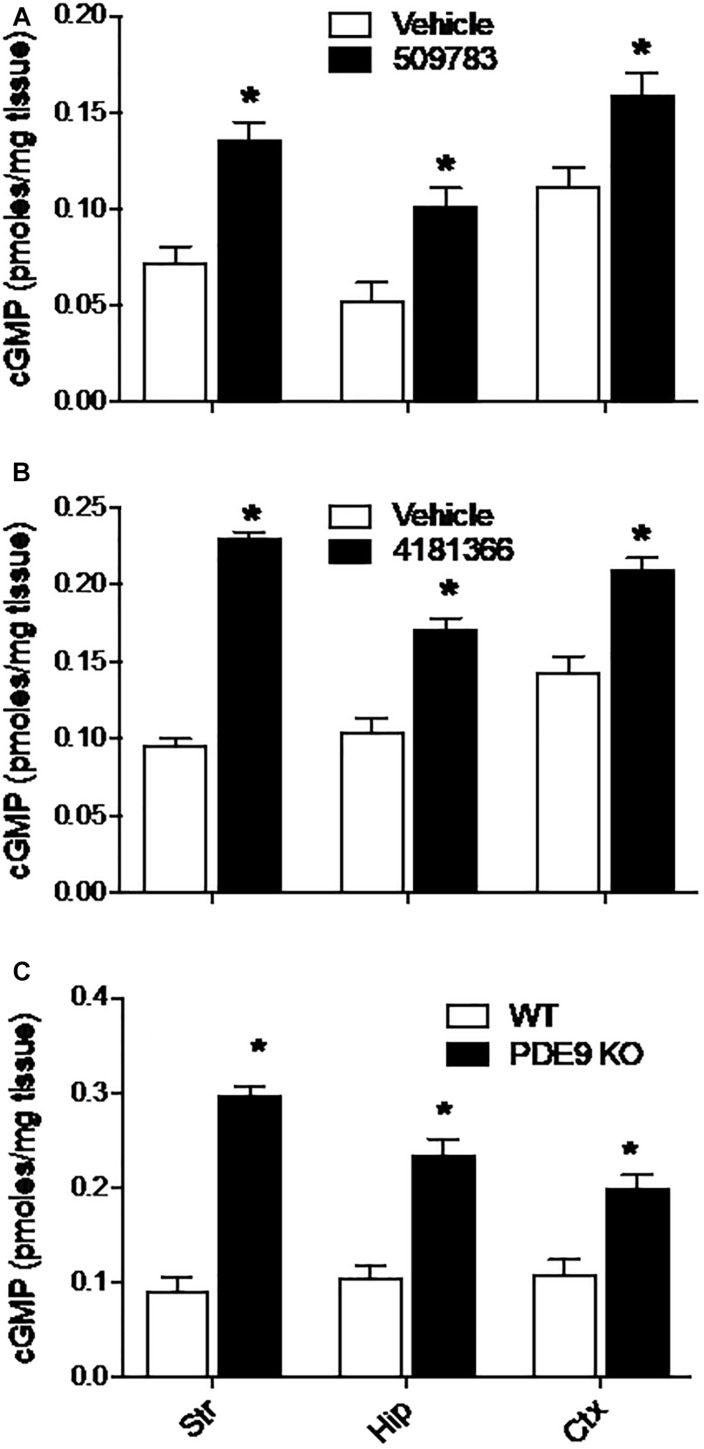
Pharmacological inhibition or genetic deletion of PDE9A elevated brain cGMP. PDE9A inhibitors PF-509783 **(A)** and PF-4181366 **(B)** administered 30 min prior to sacrifice induce significant elevations in cGMP content in striatum (Str), hippocampus (Hip), and cortex (Ctx) as compared to vehicle treated controls. PDE9A knockout mice **(C)** exhibit significant elevations in cGMP content in these same brain regions as compared to wild type littermates. At the doses tested, the effects of the PDE9A inhibitors were similar to that of PDE9A gene knock out. Results were analyzed by two-way ANOVA (Gene status or PDE9A inhibitor X brain region) followed by *post hoc* tests of the effect of gene status or treatment in each brain region, corrected for multiple comparisons. There was a significant main effect of PF-509783 [*F*(1, 24) = 50.34, *P* < 0.0001], a main effect of brain region [*F*(2, 24) = 20.17, *P* < 0.0001], but no interaction [*F*(2, 24) = 0.4869, *P* = 0.6205]. Subsequent *post hoc* tests indicated that the increase in cGMP with PF-509783 treatment was statistically significant in all brain regions (*P* < 0.05). There was also a significant main effect of PF-4181366 [*F*(1, 36) = 249.4, *P* < 0.0001], a main effect of brain region [*F*(2, 36) = 16.26, *P* < 0.0001] and an interaction [*F*(2, 36) = 15.49, *P* < 0.0001]. Subsequent *post hoc* tests indicated that the increase in cGMP with PF-4181366 treatment was statistically significant in all brain regions (*P* < 0.05). The interaction likely reflected the greater PF-4181366-induced increase in striatal cGMP relative to hippocampus and cortex. Finally, there was a significant main effect of PDE9A gene status [*F*(1, 24) = 174, *P* < 0.0001], a main effect of brain region [*F*(2, 24) = 4.758, *P* = 0.0182] and an interaction [*F*(2, 24) = 9.71, *P* = 0.0008]. Subsequent *post hoc* tests indicated that the increase in cGMP in PDE9A knock out animals compared to wild type was statistically significant in each brain region (*P* < 0.05). Again, the interaction likely reflected a greater effect of the knock out on striatal cGMP. *N* = 5 animals per treatment condition. ^∗^Significant in *post-hoc* tests at the *P* < 0.05 level.

Systemic administration of PF-4181366 to mice has previously been reported to cause a dose dependent increase in cGMP levels in striatum (Verhoest et al., 2009). Here, we investigated the effects of pharmacological PDE9A inhibition across the brain in CD-1 mice. Basal levels of cGMP varied approximately twofold across striatum, hippocampus, and cortex (Figures 1A,B). Systemic administration of PF-509783 (32 mg/kg, s.c., 30 min) or PF-4181366 (10 mg/kg, s.c., 30 min) produced significant increases in cGMP levels in each of these brain regions (Figures 1A,B, respectively). With both compounds, the increases in cGMP concentration over basal level were greater in striatum and hippocampus than in cortex.

We also examined the effect of PDE9A gene knock out on brain cGMP levels (Lee et al., 2015). In wild type mice (mixed C57BL/6J-C57BL/6N background), basal cGMP levels were comparable across cortex, hippocampus, and striatum (Figure 1C) and similar to baseline levels observed in the CD-1 mice. Compared to wildtype littermates, PDE9A KO mice (age 6–8 weeks) exhibited significantly elevated cGMP levels in cortex, hippocampus, and striatum (Figure 1C). As was observed with the PDE9A inhibitors, the magnitude of the increase in cGMP was greater in striatum and hippocampus than in cortex.

Additionally, we found much higher and variable basal cGMP levels in cerebellum than in forebrain structures. We attribute the variability to the fact that the optimal focused microwave application was directed at the forebrain regions, which yields variable and incomplete tissue fixation in the cerebellum. Both PDE9A inhibitors as well as gene knock out increased cGMP levels in cerebellum (data not shown), which is consistent with the highly expressed PDE9A mRNA in the cerebellum. Thus, while the effects of PDE9A inhibition was qualitatively similar in cerebellum to those observed in forebrain, this data was not included in the analyses.

The results described above indicate that PDE9A regulates an actively turning over pool(s) of cGMP in these brain regions. We investigated the nature of the signaling cascade(s) that drive this cGMP formation, focusing our analyses on the striatum and hippocampus.

### Effects of Glutamate Receptor Modulators on PDE9A-Regulated cGMP

In the brain, NO is a diffusible messenger formed in response to excitatory glutamate synaptic transmission by Ca^2+^ activation of nNOS (Bredt et al., 1990). We investigated whether PDE9A regulates a cGMP pool downstream of glutamate receptor signaling by determining the effects of AMPA and NMDA receptor antagonism on the increase in cGMP induced by PDE9A inhibition. The rationale was that blockade of ionotropic glutamate receptors would decrease nNOS activation and NO formation to reduce the drive on cGMP synthesis. If PDE9A were involved in degrading cGMP in this pool, this would be reflected in reduced cGMP accumulation after PDE9A inhibition in the presence of the glutamate receptor antagonists.

**FIGURE 2:**
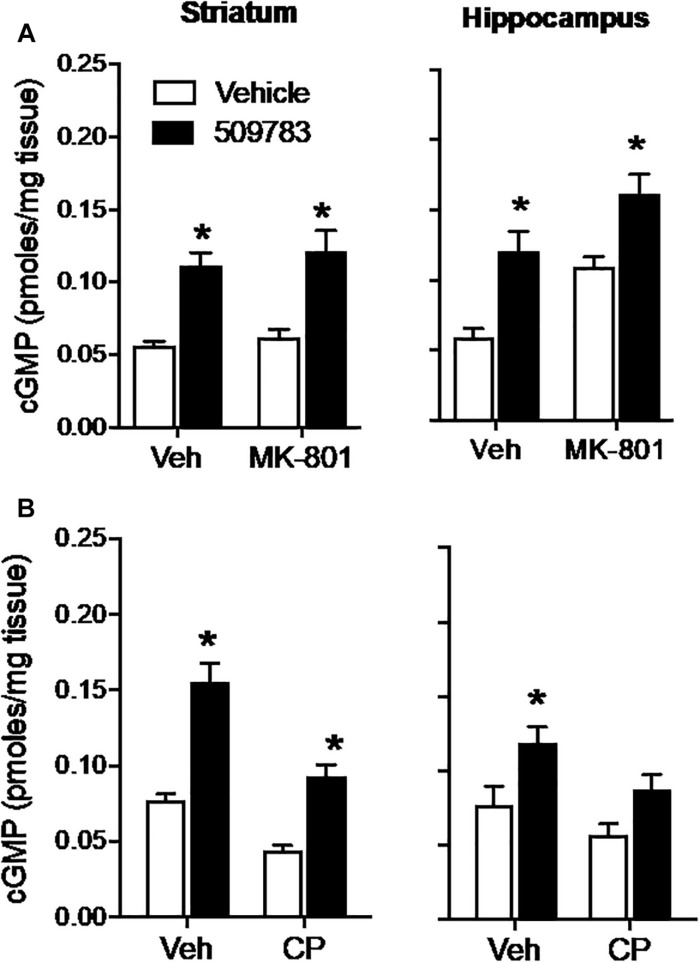
Inhibition of glutamate receptors only slightly attenuated the increase in cGMP induced by PDE9A inhibition. The NMDA receptor antagonist MK-801 (1.0 mg/kg) or the AMPA receptor antagonist CP-465022 (CP, 10 mg/kg) were administered 20 min before administration of PF-509783 (32 mg/kg) and animals were sacrificed 30 min later. MK-801 alone caused an increase in hippocampal cGMP **(A)**, whereas CP-465022 reduced cGMP in both brain regions **(B)**. PF-509783 elevated cGMP levels in both brain regions in the absence or presence of the glutamate receptor antagonists. Results were analyzed by two-way ANOVA (PDE9A inhibitor X receptor antagonist) in each brain region followed by *post hoc* tests of the effect of the PDE9A inhibitor after vehicle or drug, corrected for multiple comparisons. MK-801 **(A)**. In the striatum, there was a significant main effect of PF-509783 [*F*(1, 20) = 41.96, *P* < 0.0001), but not a main effect of MK-801 [*F*(2, 20) = 0.769, *P* = 0.391) or an interaction [*F*(2, 20) = 0.032, *P* = 0.8596). Subsequent *post hoc* tests indicated that the cGMP increase following PF-509783 administration was statistically significant in the presence of either vehicle or MK-801. In the hippocampus, there was a significant main effect of PF-509783 [*F*(1, 20) = 26.62, *P* < 0.0001], and a main effect of MK-801 [*F*(1, 20) = 17.54, *P* = 0.0005] but not an interaction [*F*(1, 20) = 0.1806, *P* = 0.6754]. *Post hoc* tests indicated the cGMP increase with PF-509783 administration was statistically significant in the absence or presence of MK-801 (*P* < 0.05). CP-465,022 **(B)**. In the striatum, there was a significant main effect of PF-509783 [*F*(1, 20) = 70.39, *P* < 0.0001], and a main effect of CP-465022 [*F*(1, 20) = 40.82, *P* < 0.0001] but not an interaction [*F*(1, 20) = 3.703, *P* = 0.0687]. In the hippocampus, there was a significant main effect of PF-509783 [*F*(1, 18) = 12.63, *P* = 0.0023], and a main effect of CP-465022 [*F*(1, 18) = 6.379, *P* = 0.0211] but not an interaction [*F*(1, 18) = 0.3138, *P* = 0.5823]. Subsequent *post hoc* tests indicated that cGMP increase observed following PF-509783 administration was statistically significant in the presence of vehicle in both brain regions and in the presence of CP-465022 in the striatum (*P* < 0.05). There was a trend for a PF-509783-induced increase in cGMP in the presence of CP-465022 in hippocampus but this did not reach statistical significance. *N* = 5 animals per treatment condition. ^∗^Significant in *post-hoc* tests at the *P* < 0.05 level.

The NMDA receptor channel blocker MK-801 (1 mg/kg, s.c.) had no effect on basal cGMP level in striatum, yet increased cGMP in hippocampus (Figure 2A, right). PF-509783 (32 mg/kg, s.c.) administered in the presence of MK-801 or vehicle significantly increased cGMP levels in both brain regions (Figure 2A). However, there was no statistically significant PF-509783 by MK-801 interaction effect, indicating that the PDE9A inhibitor response was similar regardless of MK-801 treatment. This is reinforced by comparison of the magnitudes of the PF-509783-induced increases in cGMP in the absence or presence of MK-801 (Table 1).

AMPA receptors were blocked with CP-465022 (10 mg/kg, s.c.), which at the dose tested was reported to cause a substantial reduction in the population spike amplitude in CA1 after stimulation of the Schaeffer collateral/commissural pathway in urethane-anesthetized rats and completely inhibit pentylenetetrazole-induced seizures in mice (Menniti et al., 2003). CP-465022 reduced basal cGMP level in both striatum and hippocampus (Figure 2B). When administered with CP-465022, the PDE9 inhibitor increased cGMP levels in both brain regions, however, the effect only reached statistical significance in the striatum. As presented in Table 1, the absolute increase in tissue cGMP produced by PF-509783 was slightly smaller in the presence of CP-465022 than in the vehicle treated groups. However, the PF-509783 by CP-465022 interaction terms in the 2 way ANOVAs were not significant suggesting that inhibition of AMPA receptors did not specifically block activation of cGMP synthesis in the pool regulated by PDE9A. Repeating this experiment yielded similar results and the same conclusion.

**FIGURE 3:**
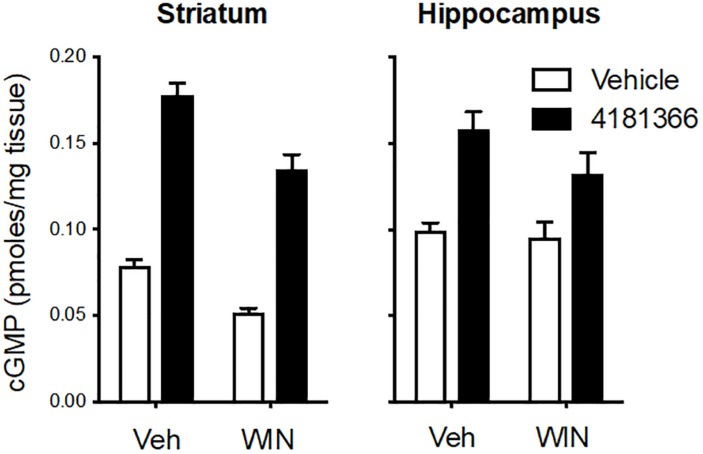
Inhibition of CB1 receptors only slightly attenuated the increase in cGMP induced by PDE9A inhibition. The CB1 receptor antagonist WIN 55,212 (WIN, 10 mg/kg, i.p.) was administered 20 min before administration of PF-4181366 (10 mg/kg) and animals were sacrificed 30 min later. WIN alone caused a decrease in cGMP in striatum but had no effect in hippocampus. PF-4181366 elevated cGMP levels in both brain regions in the absence or presence of WIN. Results were analyzed by two-way ANOVA (PF-4181366 × WIN) in both brain regions followed by *post hoc* tests of the effect of PF-4181366 after vehicle or WIN corrected for multiple comparisons. In the striatum, there was a significant main effect of PF-4181366 [*F*(1, 20) = 257.1, *P* < 0.0001] and a main effect of WIN [*F*(12, 20) = 36.62, *P* < 0.0001], but there was no PF-4181366 × WIN interaction [*F*(1, 20) = 1.97, *P* = 0.176]. Subsequent *post hoc* tests indicated that the cGMP increase following PF-4181366 administration was statistically significant in the presence of either vehicle or WIN. In the hippocampus, there was a significant main effect of PF-4181366 [*F*(1, 20) = 25.41, *P* < 0.0001] but no main effect of WIN [*F*(1, 20) = 2.48, *P* = 0.191] and no interaction [*F*(1, 20) = 1.375, *P* = 0.255]. *Post hoc* tests indicated the cGMP increase with PF-4181366 administration was statistically significant in the absence or presence of WIN (*P* < 0.05).

Glutamatergic neurotransmission is mediated by 3 classes of ionotropic receptors and 5 classes of metabotropic receptors. To more broadly assess a potential role of glutamatergic signaling in the maintenance of the PDE9 sensitive pool of cGMP we took advantage of the well-established regulation of glutamate release by inhibitory presynaptic CB1 receptors on cortical afferents in the striatum (Gerdeman and Lovinger, 2001; Covey et al., 2017). WIN 55212 alone caused a decrease in cGMP in striatum but had no effect in hippocampus (Figure 3). PF-4181366 elevated cGMP levels in both brain regions in the absence or presence of WIN 55212. Importantly, there was no statistical interaction between the two treatments used in combination in either brain region (Figure 3, legend). While the results are consistent with a role for glutamatergic signaling in the maintenance of a pool of striatal cGMP (Gerdeman and Lovinger, 2001), this pool is not subject to regulation by PDE9. WIN 55212 had no effect on basal or the PDE9 inhibitor-induced cGMP signal in the hippocampus.

### Effects of Dopamine Receptor Modulators on PDE9A-Regulated cGMP

Dopamine signaling activates NO-dependent cGMP signaling in the striatum to regulate the excitability of medium spiny neurons (West, 2004; West and Tseng, 2011). Furthermore, PDE9A inhibition modulates the activity of dopaminergic drugs in some rodent models (Kleiman et al., 2012). Thus, we investigated the involvement of PDE9A in regulation of DA receptor-modulated cGMP signaling. The strategy was analogous to that described above. In this case, we determined the effects of dopamine D1 and D2 receptor antagonists on the increase in cGMP induced by PDE9A inhibition.

**FIGURE 4:**
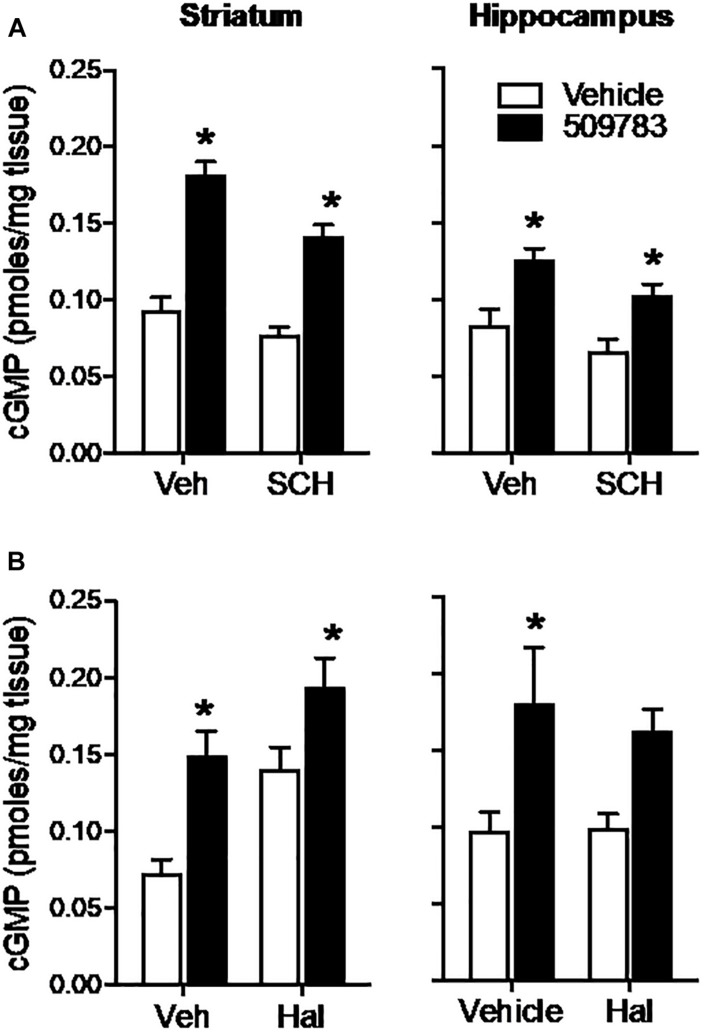
Inhibition of dopamine receptors only slightly attenuated the increase in cGMP induced by PDE9A inhibition. The D1 antagonist SCH-23390 (SCH, 1.0 mg/kg) was administered 20 min prior to PF-509783 (32 mg/kg) while the D2 receptor antagonist was administered 5 min post PF. Sacrifice was 30 min post PF. SCH-23390 alone caused a slight decrease in both striatal and hippocampal cGMP **(A)**, whereas haloperidol caused a substantial increase in striatal cGMP **(B)**. PF-509783 elevated cGMP levels in both brain regions in the absence or presence of the dopamine receptor antagonists. SCH-23390 **(A)**. In the striatum, there was a significant main effect of PF-509783 [*F*(1, 19) = 95.33, *P* < 0.0001], and a main effect of SCH-23390 [*F*(1, 19) = 12.53, *P* = 0.0022] but not an interaction [*F*(1, 19) = 2.481, *P* = 0.1318]. Similarly, in hippocampus, there was a significant main effect of PF-509783 [*F*(1, 18) = 21.11, *P* = 0.0002], and a main effect of SCH-23390 [*F*(1, 18) = 5.532, *P* = 0.0303] but not an interaction [*F*(1, 18) = 0.1476, *P* = 0.7054]. Subsequent *post hoc* tests indicated that the cGMP increase observed following PF-509783 administration was statistically significant in the presence of either vehicle or SCH-23390 in both brain regions (*P* < 0.05). Haloperidol **(B)**. In the striatum, there was a significant main effect of PF-509783 [*F*(1, 20) = 18.72, *P* = 0.0003], and a main effect of haloperidol [*F*(1, 20) = 14.27, *P* = 0.0012] but not an interaction [*F*(1, 20) = 0.6052, *P* = 0.4457]. In the hippocampus, there was a significant main effect of PF-509783 [*F*(1, 20) = 12.41, *P* = 0.0021], but not a main effect of haloperidol [*F*(1, 20) = 0.1683, *P* = 0.6860] or an interaction [*F*(1, 20) = 0.2407, *P* = 0.6290]. Subsequent *post hoc* tests indicated that cGMP increase observed following PF-509783 administration was statistically significant in the presence of vehicle in both brain regions and in the presence of haloperidol in the striatum (*P* < 0.05). *N* = 5 animals per treatment condition, except for the PF-509783 plus SCH-23390 condition, where *n* = 4. ^∗^Significant in *post-hoc* tests at the *P* < 0.05 level.

The D1 receptor antagonist SCH-23390 (1 mg/kg, s.c.) significantly reduced basal cGMP level in both striatum and hippocampus (Figures 4A,B; see Figure 3 legend for details of the statistical analyses). PF-509783 (32 mg/kg, s.c.) administered with SCH-23390 or vehicle significantly increased cGMP levels in both brain regions, with the effect of PF-509783 only slightly reduced by the D1 antagonist (Table 1). However, no significant SCH-23390 by PF509783 interaction was observed.

The D2 receptor antagonist haloperidol (0.32 mg/kg, s.c.) when dosed alone, increased cGMP levels in the striatum, but not in the hippocampus (Figure 4). PF-509783 (32 mg/kg, s.c.) administered in conjunction with haloperidol or vehicle increased cGMP levels in both brain regions, but the effect did not reach statistical significance in the hippocampus. The increase in cGMP caused by PF-509783 was slightly reduced by the D2 antagonist in both regions (Table 1). However, no significant haloperidol by PF-509783 interaction was observed.

The results summarized above indicate that dopamine receptor antagonists have little effect upon the efficacy of PDE9A inhibition to increase cGMP. These data suggest that PDE9A does not regulate the major cGMP pool modulated by dopamine receptor signaling.

### Effects of Genetic Deletion of NOS on PDE9A-Regulated cGMP

In summary of the above results, PDE9A inhibition caused increases in cGMP in striatum and hippocampus that were largely unaffected by inhibition of either glutamate or dopamine signaling. Given that both glutamate and dopamine receptor activation modulate NO formation, the lack of effects of these antagonists on the response to PF-509783 suggested that the primary pool(s) of cGMP regulated by PDE9A may not be downstream of NO signaling. We tested this possibility directly by determining the effects of genetic deletions of NOS on the increase in cGMP caused by PDE9A inhibition. In these experiments, we used PF-4181366 to inhibit PDE9A in nNOS, eNOS, and iNOS, knock out mice.

**FIGURE 5:**
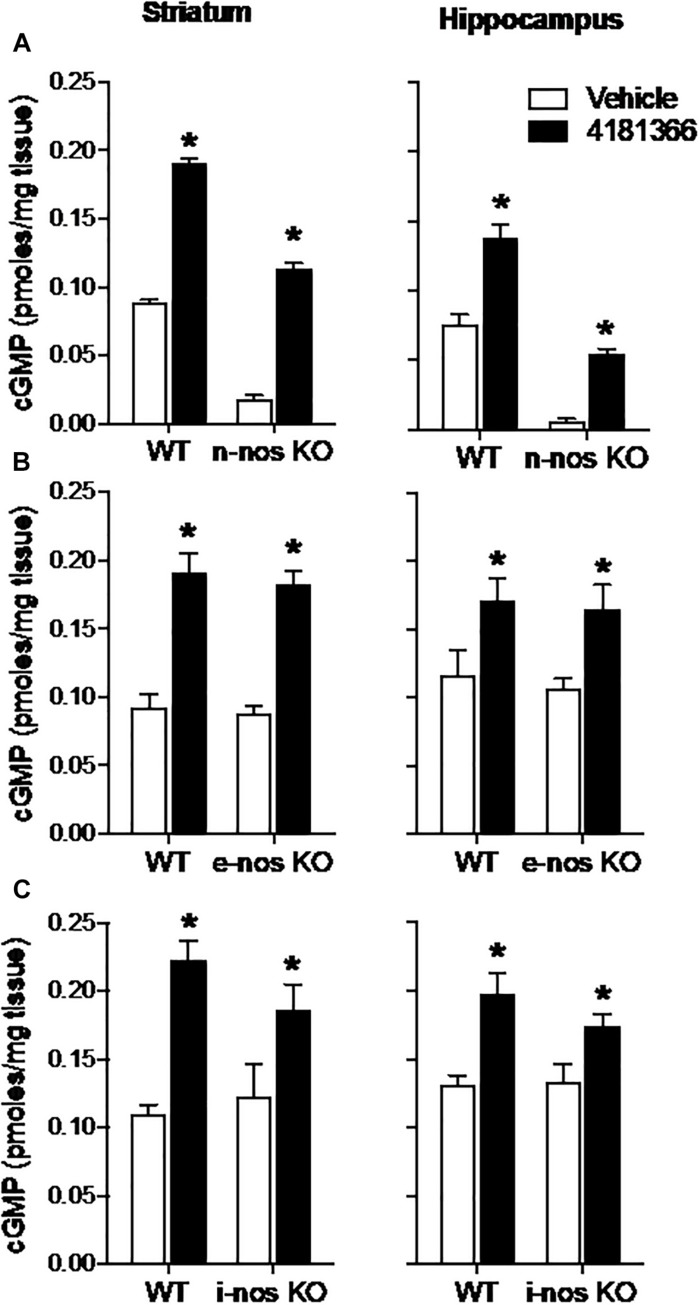
Genetic deletion of nitric oxide synthase (NOS) has no effect on PDE9A inhibitor-induced increase in cGMP. PF-4181366, administered 30 min prior to sacrifice, significantly elevated cGMP content in striatum (Str) and hippocampus (Hip) of nNOS **(A)**, eNOS **(B)**, or iNOS **(C)** knockout mice. Results were analyzed by two-way ANOVA (PDE9A inhibitor X gene status) in each brain region followed by *post hoc* tests of the effect of the PDE9A inhibitor in each strain, corrected for multiple comparisons. nNOS **(A)**. In the striatum, there was a significant main effect of PF-4181366 [*F*(1, 20) = 1297, *P* < 0.0001], and a main effect of nNOS knock out [*F*(1, 20) = 718.3, *P* < 0.0001] but not an interaction [*F*(1, 20) = 1.471, *P* = 0.2394]. Similarly, in the hippocampus, there was a significant main effect of PF-509783 [*F*(1, 19) = 82.01, *P* < 0.0001], and a main effect of strain [*F*(1, 19) = 155.7, *P* < 0.0001] but not an interaction [*F*(1, 19) = 1.437, *P* = 0.2453]. Subsequent *post hoc* tests indicated that the cGMP increase observed following PF-4181366 administration was statistically significant in both regions of wild type and nNOS knockout animals (*P* < 0.05). eNOS **(B)**. In the striatum, there was a significant main effect of PF-4181366 [*F*(1, 20) = 88.38, *P* < 0.0001], but not a main effect of eNOS knock out [*F*(1, 20) = 0.4332, *P* = 0.5179] or interaction [*F*(1, 20) = 0.03493, *P* = 0.8536]. In the hippocampus, there was a significant main effect of PF-4181366 [*F*(1, 20) = 13.44, *P* = 0.0015], but not a main effect of eNOS knock out [*F*(1, 20) = 0.2632, *P* = 0.6136] or interaction [*F*(1, 20) = 0.01823, *P* = 0.8940]. Subsequent *post hoc* tests indicated that the cGMP increase observed following PF-4181366 administration was statistically significant in both regions of wild type and e-nos knockout animals (*P* < 0.05). iNOS **(C)**. In the striatum, there was a significant main effect of PF-4181366 [*F*(1, 20) = 28.11, *P* < 0.0001], but not a main effect of strain [*F*(1, 20) = 0.5026, *P* = 0.4865] or interaction [*F*(1, 20) = 2.125, *P* = 0.1604]. In the hippocampus, there was a significant main effect of PF-4181366 [*F*(1, 20) = 21.59, *P* = 0.0002], but not a main effect of strain [*F*(1, 20) = 0.8851, *P* = 0.3580] or interaction [*F*(1, 20) = 1.178, *P* = 0.2906]. Subsequent *post hoc* tests indicated that the cGMP increase observed following PF-4181366 administration was statistically significant in both regions of wild type and i-nos knockout animals (*P* < 0.05). *N* = 5 animals per group. ^∗^Significant in *post-hoc* tests at the *P* < 0.05 level.

The basal levels of cGMP were reduced by 80–90% in striatum and hippocampus in nNOS knock out mice as compared to wild type mice (Figure 5A). This suggests that nNOS signaling cascades predominantly drive the synthesis of basal cGMP pools in these brain regions. Despite this observation, PF-4181366 still significantly increased cGMP levels in both brain regions in the nNOS knock out mice and the magnitudes of these increases were similar to those in wild type mice (Table 1). These data clearly indicate that the PDE9A-regulated cGMP pool is not driven by nNOS signaling.

Genetic deletion of eNOS (Figure 5B) or iNOS (Figure 5C) had no effect on basal cGMP levels compared to respective wild type controls, supporting the suggestion that nNOS is the major source of basal NO-stimulated cGMP synthesis in brain. Furthermore, knock out of eNOS or iNOS did not attenuate the increase in cGMP induced by PF-4181366 (Figures 5B,C and Table 1). This indicates that these NOS isoforms likely do not contribute directly to signaling cascades maintaining the pool of cGMP regulated by PDE9A.

### Effects of PDE1B and PDE10A Gene Knock Out on PDE9A-Regulated cGMP

The results presented above indicate that PDE9A regulates a pool(s) of cGMP independent of NO signaling. We next investigated the extent to which this PDE9A-regulated cGMP pool is compartmentalized by determining the effect of PDE10A and PDE1B gene deletion on the cGMP response to PDE9A inhibition. PDE10A is highly expressed in striatum (Seeger et al., 2003) and has been shown to regulate a cGMP pool downstream of nNOS signaling (Siuciak et al., 2006a; Padovan-Neto et al., 2015). PDE1B is also highly expressed in striatum, as well as dentate gyrus within the hippocampal complex (Reed et al., 1998). PDE1B is a dual substrate phosphodiesterase, but there are no published data indicating that the enzyme regulates cGMP pools in either brain region. We investigated whether the PDE9A-regulated cGMP pool is accessible to degradation by PDE10A or PDE1B by determining whether gene knock out of these PDEs affected the cGMP response to PDE9A inhibition. If these PDEs accessed the PDE9A-regulated cGMP pool, then deletion of these genes would be predicted to increase the cGMP response to PDE9A inhibition. For these experiments, we used PF-509783 in PDE1B (Siuciak et al., 2007) or PDE10A (Siuciak et al., 2006b) knock out mice.

**FIGURE 6:**
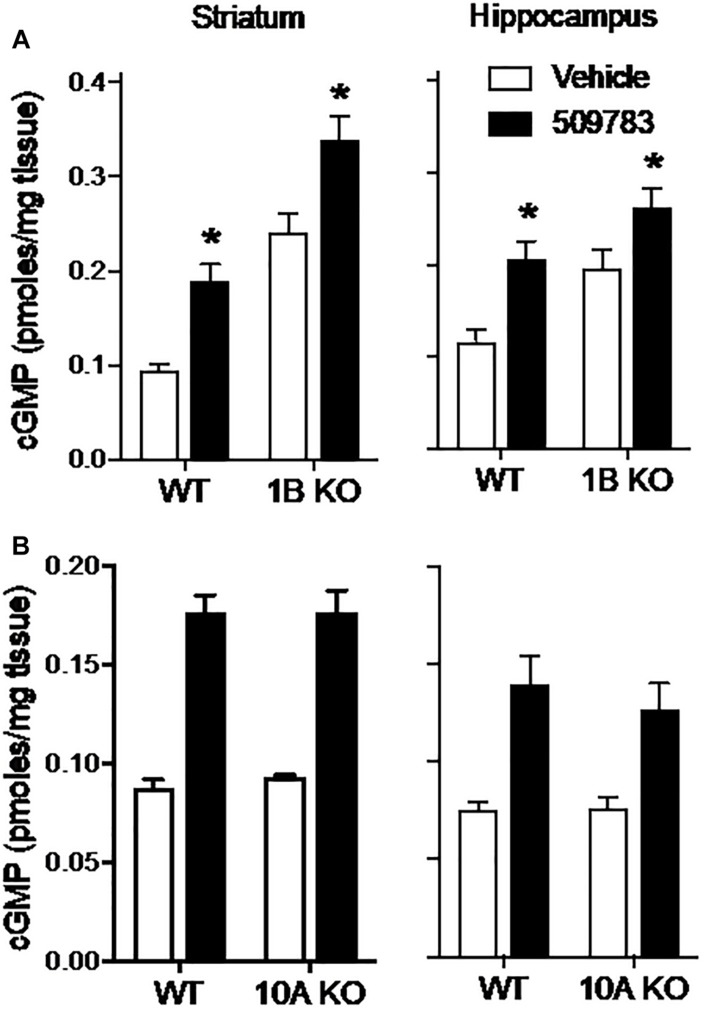
Genetic deletion of PDE1B or PDE10A did not effect increases in cGMP induced by pharmacological inhibition of PDE9A. PF-509783, administered 30 min prior to sacrifice, significantly elevated striatal (Str) and hippocampal (Hip) cGMP content in PDE1B **(A)** and PDE10A **(B)** knockout mice. Results were analyzed by two-way ANOVA (PDE9A inhibitor X gene status) in each brain region followed by *post hoc* tests of the effect of the PDE9A inhibitor in each strain, corrected for multiple comparisons. PDE1B knock out **(A)**. In the striatum, there was a significant main effect of PF-509783 [*F*(1, 16) = 28.04, *P* < 0.0001], and a main effect of PDE1B knock out [*F*(1, 16) = 66.73, *P* < 0.0001] but not an interaction [*F*(1, 16) = 0.005939, *P* = 0.9395]. In the hippocampus, there was a significant main effect of PF-509783 [*F*(1, 15) = 19.58, *P* = 0.0005], and a main effect of strain [*F*(1, 15) = 14.71, *P* = 0.0016] but not an interaction [*F*(1, 15) = 0.4524, *P* = 0.5114]. Subsequent *post hoc* tests indicated that the cGMP increase observed following PF-509783 administration was statistically significant in both regions of wild type and PDE1b knockout animals (*P* < 0.05). PDE10A knock out **(B)**. In the striatum, there was a significant main effect of PF-509783 [*F*(1, 20) = 139.9, *P* < 0.0001], but not a main effect of PDE10A knock out [*F*(1, 20) = 0.1588, *P* = 0.6945] or interaction [*F*(1, 20) = 0.109, *P* = 0.7447]. In the hippocampus, there was a significant main effect of PF-509783 [*F*(1, 20) = 33.09, *P* < 0.0001], but not a main effect of PDE10A knock out [*F*(1, 20) = 0.3876, *P* = 0.5406] or interaction [*F*(1, 20) = 0.4521, *P* = 0.5091]. Subsequent *post hoc* tests indicated that the cGMP increase observed following PF-509783 administration was statistically significant in both regions of wild type and PDE10A knockout animals (*P* < 0.05). ^∗^Significant in *post-hoc* tests at the *P* < 0.05 level.

PDE1B knock out resulted in substantial increases in basal cGMP levels in both the striatum and hippocampus (Figure 6A). PF-509783 caused a further significant cGMP increase in both brain regions. However, these increases were similar in both PDE1B knock out mice and wild type mice (Table 1), with no significant PF-509783 by PDE1B knock out interaction (see Figure 6 legend for details of the statistical analyses).

PDE10A knock out had no effect on basal cGMP levels in either striatum or hippocampus (Figure 6B). This lack of effect of the knock out in striatum is in sharp contrast to the robust increase in cGMP induced in this region by acute administration of a PDE10A inhibitor (Schmidt et al., 2008), implying induction of compensatory mechanisms in the knock out. PF-509783 caused significant increases in cGMP in both brain regions that were of similar magnitude in the PDE10A knock out and wild type mice (Figure 6B and Table 1). There was no significant PF-509783 by PDE10A knock out interaction.

In summary, the data presented in Figure 6 indicate that the PDE9A-regulated cGMP pool is confined to a discreet compartment that does not overlap with PDE1B- or PDE10A-regulated pools.

## Discussion

mRNA for the cGMP-specific PDE9A is expressed in diverse neuronal populations throughout the brain. These include cortical pyramidal neurons, hippocampal pyramidal neurons, striatal medium spiny neurons, and cerebellar Purkinje neurons (Andreeva et al., 2001; van Staveren et al., 2002; van Staveren and Markerink-van Ittersum, 2005). However, the abundance of PDE9A message is notably low in comparison to other cGMP PDEs such as PDE1, PDE2A, and PDE10A (Lakics et al., 2010; Kelly, 2012). It has also been difficult to quantify PDE9A protein levels in brain (Kleiman et al., 2012; Patel et al., 2018), which may also be due to low abundance and again is in contrast to other cGMP PDEs. The low levels of abundance notwithstanding, we show here that PDE9A inhibition induces an increase in tissue cGMP levels in each of these brain regions where message is detected. In forebrain, the effect of PDE9A inhibition on cGMP concentration is greatest in striatum and least in cortex, both in terms of absolute increase over basal level as well as fold increase over basal. In cerebellum, pharmacological PDE9A inhibition and gene knock out also increased cGMP content, although this effect was not well quantified in our studies. These observations indicate that PDE9A regulates an actively turning over cGMP signaling pool across the diverse brain regions where the enzyme is expressed.

The novel finding of the present study is that PDE9A regulates a cGMP signaling cascade in the brain that is independent of NO signaling. NO activation of sGC is the canonical pathway in brain to induce cGMP synthesis and down-stream signaling (Bredt et al., 1990; Garthwaite and Boulton, 1995; Garthwaite, 2008; West and Tseng, 2011). Most widely studied has been glutamate receptor activation of NO/cGMP signaling, in particular by NMDA receptors. In this signaling cascade, NMDA receptors and Ca^2+^-activated nNOS are brought into proximity in the postsynaptic density through association with postsynaptic scaffolding proteins. Ca^2+^ influx through NMDA receptors activates NO formation by nNOS to stimulate cGMP synthesis and signaling that mediates a very broad range of NMDA receptor regulated functions throughout the brain and spinal cord. These include aspects of synaptic plasticity (Brenman and Bredt, 1997). PDE9A is expressed in glutamatergic pyramidal neurons of cortex and hippocampus, neuronal types for which postsynaptic NMDA receptor/nNOS coupling is prominent. nNOS activation of cGMP synthesis also plays a role in dopaminergic signaling in the striatum (West, 2004; West and Tseng, 2011). In this brain region, dopamine receptor activity regulates nNOS-dependent cGMP accumulation. NO diffuses into medium spiny neurons to stimulate cGMP synthesis, where the second messenger regulates excitability (Padovan-Neto et al., 2015). PDE9A is expressed in majority of medium spiny neurons, which comprise more than 90% of the neurons in striatum. Nonetheless, while pharmacological inhibition of glutamatergic or dopaminergic signaling did impact basal cGMP concentrations in the striatum and hippocampus, these manipulations had little or no effect on PDE9A inhibitor-induced increases in cGMP levels in either brain region. These results are consistent with the established role of nNOS in the signaling cascades of both transmitter systems but indicate that the pool of cGMP regulated by PDE9A is not downstream of nNOS.

Several interesting observations in the course of these experiments deserve comment. Notably, while MK-801 had no effect on cGMP in striatum, the NMDA receptor channel blocker increased cGMP levels in hippocampus. This may have resulted from an increase in hippocampal network activity caused by this channel blocking NMDA receptor antagonist. There is an extensive literature demonstrating that such compounds alter the activities of both local and larger-scale pyramidal neuron networks, as indicated by changes in the frequencies of network oscillations (Hunt and Kasicki, 2013). One well-documented effect is to increase the frequency and power of high frequency gamma oscillations that reflect increased activity within local pyramidal neuron networks. Conversely, AMPA receptor inhibition with CP-465022, which would be expected to decrease such network activity (Menniti et al., 2003), caused a decrease in cGMP levels in both the hippocampus and striatum. The CB1 agonist WIN 55212 produced the predicted reduction in striatal cGMP consistent with the effect of AMPA receptor blockade but did not affect baseline concentrations of cGMP in the hippocampus. Although speculative, the latter may be consistent with recent reports that CB1 receptor activation can have opposing effects on distinct interneuron populations regulating glutamatergic activity in the hippocampus (Friend et al., 2019). Dopamine D1 receptor inhibition with SCH-23390 decreased cGMP in both brain regions, whereas haloperidol increased cGMP in striatum but not hippocampus. These regional differences are consistent with D1 signaling in both brain regions but robust D2 signaling only in striatum.

Prompted by the above observations, we investigated directly whether nNOS signaling drives cGMP synthesis of the PDE9A-regulated cGMP pool. Consistent with the results of our pharmacological studies, we found that genetic knock out of nNOS had no effect on the ability of a PDE9A inhibitor to increase cGMP in hippocampus or striatum. nNOS knock out did substantially and significantly decrease cGMP levels in both striatum and hippocampus, consistent with active nNOS-regulated cGMP signaling in both brain regions. In other experiments, we observed that nNOS knock out completely abrogates the effect of PDE10A inhibitors to increase striatal cGMP (Siuciak et al., 2006a; Padovan-Neto et al., 2015). There is also evidence that NO arising from eNOS activation can regulate synaptic plasticity (Dinerman et al., 1994; Hopper and Garthwaite, 2006). Thus, we extended our line of investigation but found that knock out of eNOS or iNOS also did not occlude the PDE9A inhibitor-induced increase in striatal or hippocampal cGMP levels. These results further confirmed that PDE9A regulates an active cGMP signaling cascade that is not downstream of NO signaling.

The intracellular compartmentalization of cyclic nucleotide signaling is attributed in part to the action of phosphodiesterases (Houslay, 2010; Epstein, 2017). While the results described above indicate that the PDE9A-regulated cGMP pool is independent of NO-driven cGMP pools, it was of interest to determine whether these various cGMP pools comingle, particularly after phosphodiesterase inhibition. We observed that genetic deletion of two other cGMP PDEs highly expressed in brain, PDE1B and PDE10A, had no effect on the increase in cGMP induced by PDE9A inhibition. Thus, the PDE9A-regulated cGMP pool is not only independent of pools regulated by these phosphodiesterases, it also appears to be highly segregated even in the presence of the elevated concentrations of cGMP produced by the absence of PDE1B.

It is also noteworthy that the PDE9A inhibitor-induced increases in cGMP were of similar magnitudes to those after PDE9A gene deletion. This implies the increase in cGMP does not trigger long-term compensatory feedback on synthesis or degradation within this pathway. PDE1B knock out also caused a modest increase in cGMP, whereas PDE10A knock out caused no such change. Thus, functional compensation for long term PDE inhibition appears to be pathway specific. It is also evident from our data that there must be other mechanisms for disposal of the PDE9A-regulated cGMP pool, given that the increases after PDE9A inhibition are limited. Cellular extrusion could serve as a mechanism for elimination of intracellular cGMP (Ritter et al., 2005) and so could account for the limits on the increase in tissue cGMP levels after PDE9A inhibition we observed. Consistent with such a mechanism, PDE9A inhibition causes increases in cGMP levels in cerebrospinal fluid in rodents, non-human primates, and humans (Schmidt et al., 2009; Kleiman et al., 2012; Boland et al., 2017). Cyclic nucleotides extruded from cells may have extracellular signaling functions (Rosenberg, 1992; Hofer and Lefkimmiatis, 2007), including a role for extracellular cGMP in cognition (Erceg et al., 2005). This may be another aspect of the cGMP signaling cascade regulated by PDE9A.

## Summary

The results of our studies indicate that PDE9A regulates a cGMP signaling cascade that is active throughout the brain. Contrary to assumptions (Hutson et al., 2011; Kleiman et al., 2012; Kroker et al., 2014; Su et al., 2016), this signaling cascade is independent of canonical nNOS signaling pathways or of other NO-dependent signaling mechanisms. Given that PDE9A inhibition has been found to have a number of significant neurophysiological effects and impacts cognitive function in preclinical animal models, it is of considerable interest to further investigate and characterize these PDE9A regulated signaling cascades. Of immediate interest is investigation of whether PDE9A may regulate a pathway downstream of a natriuretic peptide receptor/pGC dependent signaling mechanism in brain. It has recently been shown that PDE9A in heart exclusively regulates cGMP signaling downstream of atrial natriuretic peptide signaling, whereas PDE5A independently regulates NOS-driven cGMP signaling (Lee et al., 2015). There is a growing literature indicating that natriuretic peptide signaling may be more widespread in brain than previously appreciated (Mahinrad et al., 2016). However, the tools to investigate these signaling cascades in brain are presently limited. Linking PDE9A to natriuretic peptide signaling in brain could provide an avenue for such investigations through the use of PDE9A inhibitors as probes, much as PDE4 inhibition has informed the study of adenylyl cyclase coupled receptors. Such investigations take on added significance in light of the fact that high quality PDE9A inhibitors are now available and have entered into initial clinical trials to explore procognitive efficacy in Alzheimer’s disease and schizophrenia (Schwam et al., 2014; Wunderlich et al., 2016; Boland et al., 2017; Moschetti et al., 2018). It is critical to clearly understand the inputs and outputs of PDE9A-regulated cGMP signaling to interpret and optimize the use of PDE9A inhibitors for these and other neuropsychiatric indications and to identify other therapeutic targets within these signaling cascades.

## Data Availability

The datasets generated for this study are available on request to the corresponding author.

## Ethics Statement

Animals were handled and cared for according to the Guide for the Care and Use of Laboratory Animals (Institute of Laboratory Animal Resources, 1996) and the Pfizer Institutional Animal Care and Use Committee approved all procedures.

## Author Contributions

JH and CS designed the experiments. JH conducted the experiments. JH, FM, and CS interpreted the results and wrote the manuscript.

## Conflict of Interest Statement

All of the authors were employees of Pfizer Inc. at the time these studies were completed. However, Pfizer Inc. had no role in study design, data collection and analysis, the decision to publish, nor in the preparation of the manuscript.
